# Mechanism of dopamine binding and allosteric modulation of the human D1 dopamine receptor

**DOI:** 10.1038/s41422-021-00482-0

**Published:** 2021-03-09

**Authors:** Youwen Zhuang, Brian Krumm, Huibing Zhang, X. Edward Zhou, Yue Wang, Xi-Ping Huang, Yongfeng Liu, Xi Cheng, Yi Jiang, Hualiang Jiang, Cheng Zhang, Wei Yi, Bryan L. Roth, Yan Zhang, H. Eric Xu

**Affiliations:** 1 The CAS Key Laboratory of Receptor Research, Shanghai Institute of Materia Medica, Chinese Academy of Sciences, Shanghai, 201203 China; 2 University of Chinese Academy of Sciences, Beijing, 100049 China; 3 Department of Pharmacology, University of North Carolina at Chapel Hill, Chapel Hill, NC 27599-7365 USA; 4 Department of Biophysics and Department of Pathology of Sir Run Run Shaw Hospital, Zhejiang University School of Medicine, Hangzhou, Zhejiang 310058 China; 5 MOE Frontier Science Center for Brain Research and Brain-Machine Integration, Zhejiang University School of Medicine, Hangzhou, Zhejiang 310058 China; 6 Center for Cancer and Cell Biology, Program for Structural Biology, Van Andel Research Institute, Grand, Rapids MI USA; 7 State Key Laboratory of Drug Research and CAS Key Laboratory of Receptor Research, Shanghai Institute of Materia Medica, Chinese Academy of Sciences, Shanghai, 201203 China; 8 Department of Pharmacology and Chemical Biology, School of Medicine, University of Pittsburgh, Pittsburgh, PA 15213 USA; 9 Key Laboratory of Molecular Target & Clinical Pharmacology, and State Key Laboratory of Respiratory Disease, School of Pharmaceutical Sciences, Guangzhou Medical University, Guangzhou, Guangdong 511436 China; 10 Liangzhu Laboratory, Zhejiang University Medical Center, Hangzhou, Zhejiang 311121 China

**Keywords:** Cryoelectron microscopy, Single-molecule biophysics

Dear Editor,

Dopamine acts as an essential neurotransmitter whose signaling is conducted through five G protein-coupled receptors (GPCRs), dopamine D1 to D5 receptors (DRD1–DRD5).^1^ The D1-like receptors, comprising DRD1 and DRD5, primarily couple to the G_s_ family of G proteins to activate adenylyl cyclase and induce cAMP production. DRD1 is the most abundantly expressed dopamine receptor in the CNS.^1^ It is the central receptor mediating excitatory dopamine signaling in multiple dopaminergic pathways. Dysregulation of DRD1 signaling has been directly linked to Parkinson’s disease (PD), schizophrenia, and drug abuse.^1,2^ Due to its fundamental functions in human diseases, DRD1 has long been the subject of intensive drug development efforts toward the treatment of neuropsychiatric diseases.^3^ A majority of DRD1 agonists, including the SKF compounds, targets the orthosteric pocket of DRD1, but none has passed clinical trials for neuropsychiatric symptoms to date.^3^

GPCR positive allosteric modulators (PAMs) have been proposed to provide unique advantages over orthosteric agonists including greater receptor subtype selectivity, saturable therapeutic effects and the ability to maintain spatial and temporal patterns of endogenous dopamine signaling, which collectively may lead to reduced side effects.^3,4^ Multiple groups have reported that DRD1 PAMs, such as LY3154207, CID2886111, and DETQ, stimulate DRD1 signaling.^5,6^ With ongoing clinical investigation, DRD1 PAMs may offer new therapeutic opportunities for PD.^3,7^

Despite significant efforts, the structural basis of DRD1 ligand binding and allosteric regulation properties remains poorly understood, which has significantly impeded the discovery of potential DRD1-selective drugs with minimal side effects. Here, we report two structures of DRD1–G_s_ complexes activated by the endogenous ligand, dopamine, and a synthetic agonist, SKF81297, both in the presence of LY3154207, respectively (Fig. 1a, b). We used an engineered miniG_s_ (miniG_αs__DN) to assemble DRD1–G_s_ signaling complexes^8^ (Supplementary information, Fig. S1). To obtain stable DRD1–G_s_ complexes for structural studies, we co-expressed wild-type (WT) human DRD1, miniG_αs__DN, rat G_β1_ and bovine G_γ2_ in Sf9 insect cells. The complexes were prepared as described in Supplementary information and purified to homogeneity for single-particle cryo-EM studies (Supplementary information, Fig. S2). Two different DRD1 PAMs, CID2886111 and LY3154207, which bind to different sites on DRD1 as shown by prior studies,^5,6^ were added to further stabilize the dopamine-bound DRD1–G_s_ complex. The structures of DRD1–G_s_ complexed with dopamine/LY3154207 and SKF81297/LY3154207 were determined at a global resolution of 3.2 Å and 3.0 Å, respectively (Fig. 1a, b; Supplementary information, Fig. S3 and Table S1). The relatively high-resolution maps allowed us to unambiguously model most portions of DRD1 from S21 to Y348, the G_s_ heterotrimer, the orthosteric agonists, and the nanobody Nb35 (Supplementary information, Figs. S4 and S5a). In addition, in the SKF81297-bound DRD1 structure, clear density for LY3154207 was observed above intracellular loop 2 (ICL2) (Fig. 1b; Supplementary information, Figs. S4 and S5a), allowing us to define the binding pose of LY3154207 and the allosteric site. In the dopamine-bound DRD1 structure, the binding pose of LY3154207 can be defined (Fig. 1b; Supplementary information, Figs. S4 and S5a), but no density was observed for CID2886111.

**Fig. 1:**
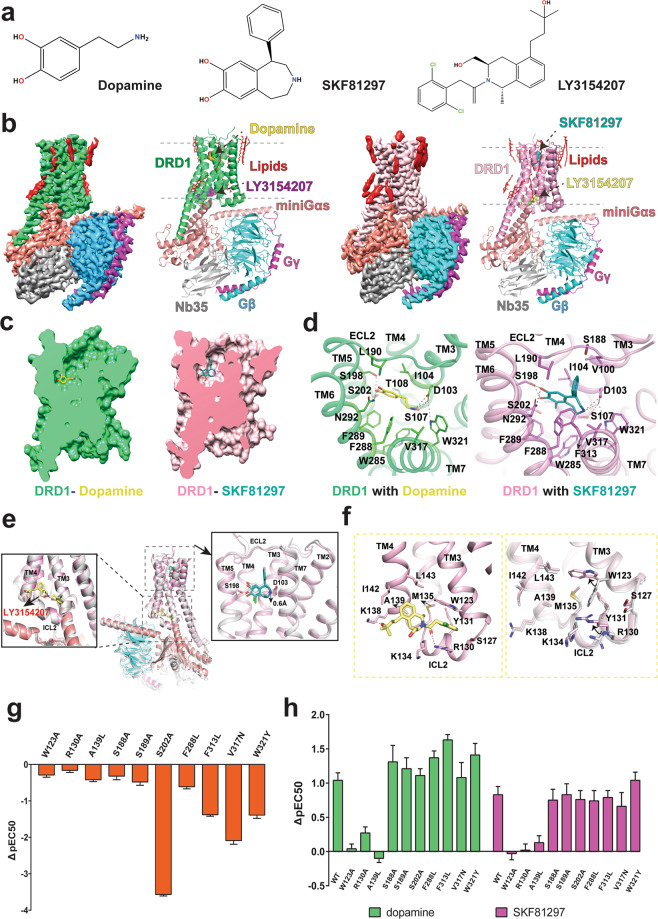
Structures of DRD1–Gs complexes. **a** Chemical structures of ligands. **b** Cryo-EM maps and structures of the DRD1–dopamine–G_s_ complex and the DRD1–SKF81297–G_s_ complex, both in the presence of LY3154207. The maps are shown at 0.045 and 0.083 thresholds for DRD1–dopamine/LY3154207–G_s_ complex and DRD1–SKF81297/LY3154207–G_s_ complex, respectively. **c** The dopamine and SKF81297 OBPs. **d** Interactions of dopamine and SKF81297 with DRD1. **e** Superposition of DRD1–SKF81297–G_s_ structures with or without LY3154207. The structure without PAM (white); the structure with PAM (DRD1, pink; SKF81297, teal; LY3154207, light yellow; G_αs_, salmon; G_β_, cyan; G_γ_, magenta). The hydrogen bond interactions are shown as black dashed line and yellow dashed line for DRD1–SKF81297 structure and DRD1–SKF81297/LY3154207 structure, respectively. **f** The binding mode of LY3154207 (left panel) and the conformational differences of the DRD1 allosteric binding site with or without LY3154207 (right panel). LY3154207 was removed in the right panel for better presentation of the alignment. **g** cAMP accumulation analysis of WT DRD1 and DRD1 mutants activated by dopamine. Data are presented as means ± SEM with a minimum of two technical replicates and *n* = 3 biological replicates. Greek letter delta (Δ) represents the difference between pEC50 values of the mutant DRD1 and  the WT receptor (ΔpEC50) . **h** Comparison of dopamine and SKF81297 in cAMP accumulation assays with WT and mutant DRD1 in the presence or absence of 30 nM LY3154207. Data are presented as means ± SEM with a minimum of two technical replicates and *n* = 3 biological replicates. ΔpEC50 is for the comparison of the pEC50s of WT or each mutant in the presence of  30 nM LY3154207, with those in the absence of LY3154207.

The overall structures of LY3154207-bound DRD1 with dopamine and SKF81297 are quite similar, with a root mean square deviation (RMSD) value of 0.6 Å for the main chain Cα atoms. However, the orthosteric binding pocket (OBP) of SKF81297 is narrower compared to that of dopamine (Fig. 1c; Supplementary information, Fig. S5b). In both structures, DRD1 adopts a canonical seven-helical transmembrane domain (TMD), the ligand-binding pockets are located at the extracellular part of the TMD and the G-protein coupling interface is located at the cytoplasmic side (Fig. 1a, b).

In the dopamine-bound DRD1 structure, dopamine occupies the OBP composed of residues from TM3, TM5–7 and capped by extracellular loop 2 (ECL2) (Fig. 1d; Supplementary information, Fig. S5a). The primary amine group forms direct ionic contacts with the carboxylate group of D103^3.32^ (superscript based on Ballesteros-Weinstein numbering rules of GPCRs), which is highly conserved in aminergic GPCRs.^9^ Such interaction is further enhanced by hydrogen bond interactions among D103^3.32^, S107^3.36^, and W321^7.43^ (Fig. 1d). The catechol moiety forms hydrogen bond interactions with S198^5.42^ and S202^5.46^ from TM5 and N292^6.55^ from TM6 (Fig. 1d). These findings agree well with the mutational results from previous studies reporting that S198^5.42^ and S202^5.46^ are pivotal for dopamine binding.^10^ In addition to the polar interaction network, hydrophobic residues I104^3.33^, L190^ECL2^, W285^6.48^, F288^6.51^, F289^6.52^ and V317^7.39^ form extensive hydrophobic interactions with dopamine to further stabilize the dopamine binding (Fig. 1d). For SKF81297, although it shares the same catechol moiety as dopamine and its benzazepine ring overlaps well with the phenylethylamine moiety of dopamine, the conformation of the catechol group of SKF81297 is slightly different from that of dopamine (Fig. 1d; Supplementary information, Fig. S5c). As a result, the catechol moiety of SKF81297 forms hydrogen bonds with S198^5.42^ but not S202^5.46^ (Supplementary information, Fig. S5c). The extra benzene group of SKF81297 occupies a small extended binding pocket (EBP) at the extracellular vestibule formed by residues V100^3.29^, L190^ECL2^, S188^ECL2^ and F313^7.35^ (Supplementary information, Fig. S5c), which contributes to its higher affinity to DRD1 than dopamine. Interestingly, the side chain of D187^ECL2^ points towards polar residues K81^2.60^ and D314^7.36^ in the SKF81297-bound DRD1 but not in the dopamine-bound DRD1, forming a potential polar interaction network (Supplementary information, Fig. S5d). The clustering of the side chains of these three polar residues leads to a narrower ligand-binding pocket for SKF81297 than that for dopamine.

To validate the structural findings in dopamine-binding pockets in DRD1, we mutated residues near the pockets and analyzed the expression levels and cAMP accumulation of these DRD1 mutants when activated by dopamine. Corresponding to the binding modes, mutations of residues D103^3.32^, S198^5.42^ and N292^6.55^ largely decreased the potency of dopamine (Supplementary information, Fig. S6 and Table S2). Furthermore, mutations of nearby residues including K81^2.61^, I104^3.33^, S107^3.36^, L190^ECL2^, S199^5.43^, F288^6.51^ and W321^7.43^ also decreased dopamine potency (Supplementary information, Figs. S6 and S7, Tables S2 and S3), supporting the binding mode of dopamine to DRD1.

DRD1 has been proposed to possess at least two different positive allosteric sites, one of these sites has been well characterized for several potent DRD1 PAMs, including DETQ and LY3154207 based on computational simulations and extensive mutagenesis data.^5,6^ In our structure, the contact pattern of LY3154207 with DRD1 is quite different from that in a previously reported simulation model of LY3154207-bound DRD1.^6^ The whole LY3154207 molecule lies in the cleft between TM3 and TM4 and right above ICL2 with a boat conformation, which is ~33 Å away from the orthosteric DRD1 pocket when measured at the Cα atoms of D103^3.32^ and Y131^ICL2^ (Fig. 1e). A similar allosteric site has also been identified in the β_2_-adrenergic receptor (β_2_AR) for a β_2_AR PAM named Cmpd-6FA^11^ (Supplementary information, Fig. S8). In the allosteric site, LY3154207 mainly forms hydrophobic and van der Waals interactions with DRD1 (Fig. 1f), which is consistent with the hydrophobic property of LY3154207. The dichlorophenyl group of LY3154207 is sandwiched by the side chains of R130^ICL2^ and W123^3.52^ to form cation–π and π–π interactions, respectively (Fig. 1f). The central tetrahydroisoquinoline (THIQ) ring of LY3154207 forms hydrophobic interactions with surrounding residues M135^ICL2^, A139^4.41^, I142^4.44^ and L143^4.45^. In addition, hydrogen bonds between LY3154207 and polar residues R130^ICL2^, K134^ICL2^ and K138^4.40^ are also observed (Fig. 1f).

To correlate the function and binding mode of LY3154207, we firstly analyzed the effects of DRD1 orthosteric site mutations on LY3154207 efficacy and potency. The G_s_-mediated cAMP accumulation results indicated that most of the mutations have minimal effects on LY3154207 binding, including residues D103^3.32^, S198^5.42^, S199^5.43^ and F288^6.51^ (Supplementary information, Fig. S6 and Table S2), which were important for dopamine and SKF81297 binding. The addition of LY3154207 increased cAMP accumulation and the potency of both dopamine and SKF81297 in WT DRD1 and DRD1 orthosteric pocket mutants (Fig. 1g, h; Supplementary information, Fig. S7 and Table S3), suggesting that orthosteric agonist and LY3154207 exert cooperative effects on G_s_ stimulation. Subsequently, we mutated most residues around the LY3154207 pocket and tested the abilities of G protein recruitment of DRD1 allosteric site mutants. The presence of LY3154207 increased potency of dopamine and SKF81297 by about one Log (Fig. 1g, h). Mutations of W123A, R130A, and A139L in the allosteric binding site nearly abolished the allosteric effects of LY3154207 on DRD1 activation potency of dopamine and SKF81297, while these mutations had modest effects on ligand binding to the orthosteric site (Fig. 1g, h; Supplementary information, Figs. S7 and S9, Tables S3 and S4). Interestingly, LY3154207 alone can activate DRD1 to a certain extent (Supplementary information, Fig. S6 and Table S2).

LY3154207 shares a high chemical similarity with DETQ. The only difference is that LY3154207 contains a longer alkyl linker between the C5 tertiary alcohol and the THIQ ring (Supplementary information, Fig. S10a). It is likely that DETQ occupies the same allosteric site as LY3154207. Supporting this hypothesis, previous studies indicated that residues W123^3.52^, R130^ICL2^ and L143^4.45^ were crucial for DETQ potency, which all directly interact with LY3154207 in the allosteric site.^5^ In addition, A139^4.41^ has been shown to play a key role in the selectivity of DETQ for DRD1 over DRD5.^5^ In our structure, A139^4.41^ in TM4 forms hydrophobic contacts with the THIQ ring of LY3154207. It is replaced by a methionine in DRD5 and the bulky side chain of methionine may preclude LY3154207 and DETQ from binding to DRD5 due to steric clash (Fig. 1f). Consistent with the hypothesis, mutation of A139L in DRD1 largely decreased LY3154207 binding (Fig. 1h; Supplementary information, Figs. S7 and S9, Tables S3 and S4). Moreover, K138^4.40^ in TM4 forms a hydrogen bond with the long stretched tertiary alcohol of LY3154207, which is likely missing for DETQ due to the shorter linker between the tertiary alcohol and the THIQ ring in DETQ (Supplementary information, Fig. S10a). Consistently, LY3154207 showed a higher affinity to DRD1 than DETQ.^6^

We have also determined a cryo-EM structure of DRD1–G_s_ complexed with SKF82197 alone.^8^ To investigate the mechanism of DRD1 allosteric modulation by LY3154207 and determine whether LY3154207 induces a different DRD1 conformation, we aligned the structures of DRD1 bound to both SKF81297 and LY3154207 and DRD1 bound to SKF81297 only. The overall DRD1 conformation in these two structures is highly similar (RMSD of 0.6 Å for Cα atoms) and the interactions between DRD1 and SKF81297 are nearly identical, but the binding poses of SKF81297 are slightly different and conformational differences can be observed in the allosteric binding pocket (Fig. 1e; Supplementary information, Fig. S10b). In the structure of DRD1 bound to LY3154207 and SKF81297, the binding pose of SKF81297 is 0.6 Å deeper compared to that of DRD1 bound to SKF81297 only, resulting in an extra hydrogen bond interaction between the *para*-hydroxyl group in the SKF81297 catechol ring and S198^5.42^ (Fig. 1e; Supplementary information, Fig. S10b), and the wider spread polar interaction may trap SKF81297 in a more stable state, thus stabilizing the active state of the receptor. Furthermore, the extensive interactions between LY3154207 and ICL2 may stabilize the α-helical structure of ICL2, which is important for receptor activation and G_s_-coupling^12^. It is likely that LY3154207 potentiates the orthosteric agonist-induced DRD1 signaling by stabilizing the active state of DRD1, which is similar to the allosteric modulation of β_2_AR by Cmpd-6FA.^11^

In conclusion, the two structures reported here uncovered the unique binding modes of dopamine and the DRD1 potent PAM LY3154207, revealed the detailed mechanism of how DRD1 is occupied by both endogenous and synthetic agonists with different chemical scaffolds, and elucidated the potential allosteric regulation of DRD1 activity. Structural comparison demonstrated the important role of the DRD1 EBP in determining its ligand potency. In addition, the structure of LY3154207 bound to DRD1 presents a first view of the DRD1 PAM-binding pocket. Together with mutagenesis results, our structures provide a framework for more efficient and subtype-selective ligand discovery targeting DRD1 for treating CNS diseases, both at the orthosteric ligand pocket and at the allosteric pocket.

## Supplementary information

Supplementary information

## Data Availability

The cryo-EM density maps have been deposited in the Electron Microscopy Data Bank: EMD-23390 (DRD1–SKF81297–LY3154207–G_s_) and EMD-23391 (DRD1–dopamine–LY3154207–G_s_). The coordinates of the structures have been deposited in the Protein Data Bank: SKF81297–LY3154207- and dopamine–LY3154207-bound DRD1–G_s_ complexes (PDB: 7LJC, 7LJD, respectively).
