# Supplementary material for: Mechanism of dopamine binding and allosteric modulation of the human D1 dopamine receptor
Source: Cell Res. 2021 Mar 9;31(5):593–6. doi: 10.1038/s41422-021-00482-0 (PMC8089099; doi:10.1038/s41422-021-00482-0)
Supplement: Supplementary file 1 — Supplementary information [file 41422_2021_482_MOESM1_ESM.pdf]

Supplementary information

Materials and Methods

Construct design

The full-length gene coding sequence of human DRD1 was synthesized with no additional mutations or loop deletions added into the gene sequence. The N-terminal of DRD1 sequence was induced with FLAG tag followed by a fragment of β_2AR N-terminal tail region (BN, hereafter) as fusion protein [1] while the C-terminal was attached with an 8×His tag to facilitate the protein expression and purification. The whole tag-attached DRD1 was then cloned into the standard pFastBac (Thermo Fisher) vector. The prolactin precursor sequence was inserted into the N-terminus before the FLAG tag as signaling peptide to assist cell membrane localization and increase expression of DRD1. A miniG$_{\alpha s}$ format of G$_{\alpha s}$ with dominant negative mutations, G226A and A366S, was engineered (miniG$_{\alpha s}$_DN) according to the previous study [2]. All the three G$_s$ protein complex components, miniG$_{\alpha s}$_DN, rat G$_{\beta 1}$ and bovine G$_{\gamma 2}$, were constructed into pFastbac vector separately.

Expression, assemble and purification of DRD1-G$_s$ complexes

Before expression, the recombinant baculoviruses containing DRD1, miniG$_{\alpha s}$_DN, G$_{\beta 1}$ and G$_{\gamma 2}$ respectively were prepared using the Bac-to-Bac baculovirus expression system (Thermo Fisher). Cell cultures were grown to a density of 4×10^6 cells/mL in ESF 921 serum-free medium (Expression Systems). For the expression of the DRD1-G$_s$ complexes,

Sf9 cells were co-infected with the four types of baculoviruses prepared at 1:1:1:1 ratio. 48 hours after infection, the cultures were harvested by centrifugation at 1300× *g* (Thermo Fisher, H12000) for 20 min and frozen at –80 °C for further usage.

For the purification of dopamine bound DRD1-miniG$_s$ complexes, cell pellets from 1 L culture were thawed at room temperature and resuspended in buffer containing 20 mM HEPES, pH 7.2, 75 mM NaCl, 5 mM $CaCl_2$, 5 mM $MgCl_2$, 10% Glycerol, 0.3 mM TECP, protease inhibitor cocktail (Bimake, 1 mL/100 mL suspension). The cell pellets were dounced to homogeneity and subsequently added with 1 mM dopamine (MCE Inhibitors) to induce complexes formation on cell membrane. Half an hour later, the cell suspension was further supplied with 10 μM LY3154207, treated with apyrase (25 mU/mL, NEB) and incubated at room temperature for another one hour. Finally, the cell membrane in suspension was solubilized directly by addition of a detergent mixture containing 0.5% (w/v) dodecyl-β-D-maltoside (DDM, Anatrace), 0.1% (w/v) cholesteryl hemisuccinate TRIS salt (CHS, Anatrace), 0.025% (w/v) digitonin (Biosynth) and supplemented with 20 μM CID2886111 and 10 μg/mL Nb35. CID2886111 is another weaker PAM of DRD1 and bind to a divergent pocket in DRD1 compared to LY3154207. The membrane was solubilized for 3 h at 4 °C before separation by ultracentrifugation. The sample was centrifuged at 100,000× *g* (Ti45, Beckman) for 45 min. The isolated supernatant was then incubated for 2 h at 4 °C directly with FLAG resin (Smart-Lifesciences) pre-equilibrated with buffer containing 20 mM HEPES, pH 7.2, 100 mM NaCl. After batch binding, FLAG

resin with immobilized protein complex was manually loaded onto a gravity flow column. Detergent was exchanged on FLAG resin by three washing steps in 20 mM HEPES, pH 7.2, 100 mM NaCl, 0.3 mM TCEP, 100 µM dopamine, 5 µM LY3154207, 10 µM CID2886111, supplied with different detergents: first 0.1% DDM, 0.02% CHS, 0.025% digitonin, then 0.02% DDM, 0.004% CHS, 0.05% digitonin, and finally 0.05% digitonin for 10 column volumes, each. The attached protein complex was then eluted in buffer containing 20 mM HEPES, pH 7.2, 100 mM NaCl, 0.3 mM TCEP, 100 µM dopamine, 5 µM LY3154207, 10 µM CID2886111, 0.05% digitonin, 200 µg/µL FLAG peptide.

For the purification of DRD1-miniG$_s$ complex bound with both SKF81297 and PAM LY3154207, all the purification processes are the same as the above for dopamine bound complex with the following exceptions. The LY3154207 compound was added into the cell suspension at the concentration of 10 µM after the first half-hour incubation with 10µM SKF81297 (Tocris) and together with the addition of apyrase (25 mU/mL, NEB). No CID2886111 was supplied in the purification. In the later purification procedures, the buffer for washing and elution were all supplemented with 5 µM SKF81297 and 5 µM LY3154207.

Released protein was further concentrated to 0.5 mL and then loaded onto a Superdex 200 10/300 GL increase column (GE Healthcare) pre-equilibrated with buffer containing 20 mM HEPES, pH 7.2, 100 mM NaCl, 0.05% digitonin, 0.1 mM TCEP, supplied with 100

μM dopamine, 5 μM LY3154207 and 10 μM CID2886111 or 5 μM SKF81297 and 5 μM LY3154207. The fractions of monomeric complex were pooled and concentrated for 20 folds (V/V), respectively. The final concentration of dopamine-LY3154207 bound D1R-G_s complex was about 10 mg/mL while concentration of SKF81297-LY3154207 bound D1R-G_s complex was 13 mg/mL. Both concentrated protein samples were used for further electron microscopy experiments.

Cryo-EM grid preparation and data collection

For the cryo-EM grid preparation, 3 μL of the purified DRD1-dopamine-LY3154207/ CID2886111-miniG_s-Nb35 complex at the concentration about 10 mg/mL, DRD1-SKF81297-LY3154207-miniG_s-Nb35 complex at the concentration of 13 mg/mL were applied individually onto glow-discharged holey carbon grids (Quantifoil, Au200 R1.2/1.3) in a Vitrobot chamber (FEI Vitrobot Mark IV). The Vitrobot chamber was set to 100% humidity at 4 °C. Excess samples were blotted for 3 s and were vitrified by plunging into liquid ethane using a Vitrobot Mark IV (Thermo Fischer Scientific). Grids were stored in liquid nitrogen for screening and data collection usage.

For DRD1-dopamine-LY3154207/CID2886111-miniG_s-Nb35 complex, cryo-EM imaging was performed on a Titan Krios at 300 kV in the Center of Cryo-Electron Microscopy, Zhejiang University (Hangzhou, China). Micrographs were recorded using a Gatan K2 Summit detector in counting mode with a pixel size of 1.014 Å using the SerialEM software

[3]. Movies were obtained at a dose rate of about 7.8 e/Å^2/s with a defocus ranging from -0.5 to -3.0 μm. The exposure time was 8 s and 40 frames were recorded per micrograph. A total of 2002 movies were collected for the dopamine-LY3154207/ CID2886111 bound DRD1-G_s complexes.

For the DRD1-SKF81297-LY3154207-miniG_s-Nb35 complex, automatic data collection was performed on a FEI Titan Krios operated at 300 kV in Cryo-Electron Microscopy Research Center, Shanghai Institute of Materia Medica, Chinese Academy of Sciences (Shanghai, China). The microscope was operated with a nominal magnification of 81,000× in counting mode, corresponding to a pixel size of 1.045 Å for the micrographs. A total of 3,607 movies for the dataset of DRD1-SKF81297-LY3154207-miniG_s-Nb35 complex were collected by a Gatan K3 Summit direct electron detector with a Gatan energy filter (operated with a slit width of 20 eV) (GIF) using the SerialEM software [3]. The images were recorded at a dose rate of about 26.7 e/Å2/s with a defocus ranging from -0.5 to -3.0 μm. The total exposure time was 3 s and intermediate frames were recorded in 0.083 s intervals, resulting in a total of 36 frames per micrograph.

Image processing and map reconstruction

For the dopamine-bound DRD1-G_s complex, image stacks were aligned using MotionCor 2.1 [4]. Contrast transfer function (CTF) parameters were estimated by Gctf v1.18 [5]. The following data processing was performed using RELION-3.0-beta2 [6]. Automated particle

selection using Gaussian blob detection produced 1,432,690 particles. The particles were subjected to reference-free 2D classification to discard fuzzy particles, resulting in 679,229 particles for further processing. The map of GPBAR-G_s complex (EMD-30344) low-pass filtered to 60 Å was used as the reference map for 3D classification, generating one well-defined subset with 279,911 particles. Further 3D classifications focusing the alignment on the complex, produced three good subsets accounting for 212,256 particles, which were subsequently subjected to 3D refinement, CTF refinement and Bayesian polishing. The final refinement generated a map with an indicated global resolution of 3.2 Å at a Fourier shell correlation of 0.143.

For DRD1-SKF81297-LY3154207-miniG_s-Nb35 complex, movie stacks were subjected to beam-induced motion correction using MotionCor2.1 [4]. Contrast transfer function parameters for each micrograph were determined by Ctffind4 [7]. The micrographs with the measured resolution worse than 4.0 Å and micrographs imaged within carbon area were discarded, generating 2,842 micrographs for further processing. Particle selection, 2D and 3D classifications were performed on a binned dataset with a pixel size of 2.09 Å using RELION-3.0-beta2 [6]. About 2000 particles were manually selected and subjected to 2D classification. Representative averages were picked as template for auto-picking. The auto-picking process produced 2,193,368 particles, which were subjected to reference-free 2D classifications to exclude fuzzy particles. The 3D density map of DRD1-SKF83959-G_s [2] low-pass filtered to 40 Å was served as initial reference map for seven rounds 3D

classifications, resulting in a single well-defined subset with 249,418 particles. Subsequent 3D refinement, CTF refinement, Bayesian polishing and DeepEnhancer [8] processing generated a map with an indicated global resolution of 3.0 Å at a Fourier shell correlation of 0.143.

Structure model building and refinement

The structure of DRD1-SK81297-G_s (PDB: 7JV5) was used as initial model for model rebuilding and refinement against the electron microscopy maps of DRD1-G_s complexes. The PDB models of LY3154207, and dopamine were generated with ChemSketch (https://www.acdlabs.com/resources /freeware/chemsketch/index.php). The initial models were docked into the electron microscopy density maps using Chimera [9] followed by iterative manual adjustment and rebuilding in COOT [10]. Real space refinement and reciprocal space refinement were performed using Phenix programs [11]. The model statistics were validated using MolProbity [12]. Structure figures were prepared in Chimera and PyMOL (https://pymol.org/2/). The final refinement statistics are provided in Supplementary information, Tables S1.

G_s Bioluminescence Resonance Energy Transfer (BRET) Recruitment Assays

To measure G protein recruitment (BRET), HEK293T cells (ATCC CRL-11268) maintained in DMEM containing 10% (v/v) dialyzed FBS, 1 IU/ml Penicillin G, and 100 μg/mL Streptomycin were passed to 10 cm dishes and co-transfected using TransIT (Mirus

Bio) in an approximate 1:2.5 ratio with DRD1 containing C-terminal *Renilla* luciferase (*R*Luc) and Venus-tagged N-terminal MiniG protein [13]. After at least 24 h, transfected cells were plated in poly-lysine coated 96-well white clear bottom cell culture plates in plating media (DMEM containing 1% (v/v) dialyzed FBS, 1 IU/mL Penicillin G, and 100 μg/mL Streptomycin) at a density of 40,000 cells in 200 μL per well and incubated overnight.

The following day, media was aspirated and cells were washed once with 60 μL of drug buffer (1× HBSS, 20 mM HEPES, pH 7.4). Then 60 μL of drug buffer was added per well and drug stimulation was performed with the addition of 15 μL of 6× drug dilution of LY3154207 in drug dilution buffer (1× HBSS, 20 mM HEPES, 0.3% (w/v) BSA, 0.03% (w/v) ascorbic acid, pH 7.4) per well and incubated at RT. After 60 min of incubation, 10 μL of the *R*Luc substrate, coelenterazine h (Promega) at final concentration of 5 μM was added per well. After an additional 5 min, 15 μL of 6× drug dilution of SKF81297 in drug dilution buffer was added to the each well, plates were read for both luminescence at 485 nm and fluorescent eYFP emission at 530 nm for 1 s per well using a Mithras LB940 (Berthold Technologies). Plates were read for multiple time points up to 30 min. The BRET ratio of eYFP/*R*Luc was calculated per well and the net BRET ratio was calculated by subtracting the eYFP/*R*Luc ratio per well from the eYFP/*R*Luc ratio in wells without Venus-tagged N-terminal MiniG protein present. The net BRET ratio was normalized to the no drug addition and plotted as a function of drug concentration using Graphpad Prism 8 (Graphpad Software Inc., San Diego, CA).

DRD1 G_S-mediated G_S-cAMP Accumulation Assay

DRD1 G_s-mediated G_s-cAMP accumulation assays with HEK293T (ATCC CRL-11268) were performed using cells transiently expressing human DRD1 and the cAMP biosensor GloSensor-22F (Promega). Cells were seeded (20,000 cells/35 µL/well) into white 384 clear-bottom, tissue culture plates in DMEM containing 1% (v/v) dialyzed fetal bovine serum (FBS). Next day, 3× drug dilutions were diluted in HBSS, 20 mM N-(2-hydroxyethyl) piperazine-N′-ethanesulfonic acid (HEPES), 0.3% (w/v) bovine serum albumin (BSA), 0.03% (w/v) ascorbic acid, pH 7.4. Media was decanted from 384 well plates and 20 µL of drug buffer (HBSS, 20 mM HEPES, pH 7.4) containing GloSensor reagent was added per well and allowed to equilibrate for at least 15 min at room temperature. Cells were then treated with 10 µL per well of 3× drug using a FLIPR (Molecular Devices). After 15 min, G_s-cAMP accumulation was read on a TriLux Microbeta (PerkinElmer) plate counter. Data were normalized to maximum G_s-cAMP accumulation by dopamine (100%). Data were analyzed using the sigmoidal log(agonist) vs. dose response function built into GraphPad Prism 8.4.

Figure preparation

The density maps were prepared in UCSF Chimera (https://www.cgl.ucsf.edu/chimera/) and and UCSF ChimeraX (https://www.cgl.ucsf.edu/chimerax/). Structural comparisons and alignments figures were prepared with PyMOL (https://pymol.org/2/).

REFERENCES

1 Xing, C. *et al.* Cryo-EM Structure of the Human Cannabinoid Receptor CB2-Gi Signaling Complex. *Cell* **180**, 645-654 (2020).

2 Zhuang, Y. *et al.* Structural insights into the human D1 and D2 dopamine receptor signaling complexes. *Cell* **184**, 931-942 (2021).

3 Mastronarde, D. N. Automated electron microscope tomography using robust prediction of specimen movements. *J Struct Biol* **152**, 36-51 (2005).

4 Zheng, S. Q. *et al.* MotionCor2: anisotropic correction of beam-induced motion for improved cryo-electron microscopy. *Nature Methods* **14**, 331-332 (2017).

5 Zhang, K. Gctf: Real-time CTF determination and correction. *J Struct Biol* **193**, 1-12 (2016).

6 Zivanov, J. *et al.* New tools for automated high-resolution cryo-EM structure determination in RELION-3. *Elife* **7**, doi:10.7554/eLife.42166 (2018).

7 Rohou, A. & Grigorieff, N. CTFFIND4: Fast and accurate defocus estimation from electron micrographs. *J Struct Biol* **192**, 216-221 (2015).

8 Sanchez-Garcia, R. *et al.* DeepEMhancer: a deep learning solution for cryo-EM volume post-processing. bioRxiv, doi:10.1101/2020.06.12.148296 (2020).

9 Pettersen, E. F. *et al.* UCSF Chimera--a visualization system for exploratory research and analysis. *J Comput Chem* **25**, 1605-1612 (2004).

10 Emsley, P. & Cowtan, K. Coot: model-building tools for molecular graphics. *Acta crystallographica. Section D, Biological crystallography* **60**, 2126-2132 (2004).

11 Adams, P. D. *et al.* PHENIX: a comprehensive Python-based system for macromolecular structure solution. *Acta crystallographica. Section D, Biological crystallography* **66**, 213-221 (2010).

12 Chen, V. B. *et al.* MolProbity: all-atom structure validation for macromolecular crystallography. *Acta crystallographica. Section D, Biological crystallography* **66**, 12-21 (2010).

13 Wan, Q. *et al.* Mini G protein probes for active G protein-coupled receptors (GPCRs) in live cells. *J. Biol. Chem.* **293**, 7466-7473 (2018).

Flag-BN-DRD1-H8

Prolactin precursor FLAG BN fusion tag

MDSKGSSQKGSRLLLLLVVSNLLLCQGVVSDYKDDDDVDMGQPGNGSAFLLAPNGSHAPDHDVTQ
QRDEASMRTLNTSAMDGTGLVVERDFSVRILTACFLSLLILSTLLGNTLVCAAVIRFRHLRSKVTNFF
VISLAVSDLLVAVLVMPWKAVAEIAGFWPFGSFCNIWVAFDIMCSTASILNLCVISVDRYWAISSPFRY
ERKMTPKAAFILISVAWTLSVLISFIPVQLSWHKAKPTSPSDGNATSLAETIDNCDSSLSRTYAISSS
VISFYIPVAIMIVTYTRIYRIAQKQIRRIAALERAAVHAKNCQTTTGNGKPVECSQPESSFKMSFKRET
KVLKTLSVIMGVFVCCWLPFFILNCILPFCGSGETQPFCIDSNTFDVFVWFGWANSSLNPIIYAFNAD
FRKAFSTLLGCYRLCPATNNAIETVSINNNGAAMFSSHHEPRGSISKECNLVYLIPHAVGSSEDLKK
EEAAGIARPLEKLSPALSVILDYDTDVSLEKIQPITQNGQHPTHHHHHHHH

8×His

miniGαs_DN

MGCLGNSKTEDQRNEEKAQREANKMIEKQLQKDKQVYRATHRLLLLGADNSGKSTIVKQMRIYHVN
SGIFETKFQVDKVNFHMFDVGAQRDERRKWIQCFNDVTAIIFVVDSSDYNRLQEALNDFKSIWNNR
WLRTISVILFLNKQDLLAEKVLAGKSKIEDYFPEFARYTTPEDATPEPGEDPRVTRAKYFIRDEFLRIST
ASGDGRHYCYPHFTCSVDTENARRIFNDCRDIIQRMHLRQYELL

H8-Gβ1

HHHHHHHHMGSLLQSELDQLRQEAEQLKNQIRDARKACADATLSQITNNIDPVGRIQMRTRRTLRG
HLAKIYAMHWGTDSRLLVSASQDGKLIIWDSYTTNKVHAIPLRSSWVMTCAYAPSGNYVACGGLDNI
CSIYNLKTREGNVRVSRELAGHTGYLSCCRFLDDNQIVTSSGDTTCALWDIETGQQTTTFTGHTGDV
MSLSLAPDTRLFVSGACDASAKLWDVREGMCRQTFTGHESDINAICFFPNGNAFATGSDDATCRLF
DLRADQELMTYSHDNIICGITSVSFSKSGRLLLAGYDDFNCNVWDALKADRAGVLAGHDNRVSCLG
VTDDGMAVATGSWDSFLKIWN

Gγ2

ASNNTASIAQARKLVEQLKMEANIDRIKVSKAAADLMAYCEAHAKEDPLLTPVPASENPFREKKFFC

Nb35

MQVQLQESGGGLVQPGGSLRLSCAASGFTFSNYKMNWVRQAPGKGLEWVSDISQSGASISYTGS
VKGRFTISRDNAKNTLYLQMNSLKPEDTAVYYCARCPAPFTRDCFDVTSTTYAYRGQGTQVTVSSH
HHHHH

Fig. S1 The amino acid sequences of DRD1-G_s complex components used in this study.

The DRD1 sequence is shown in green.

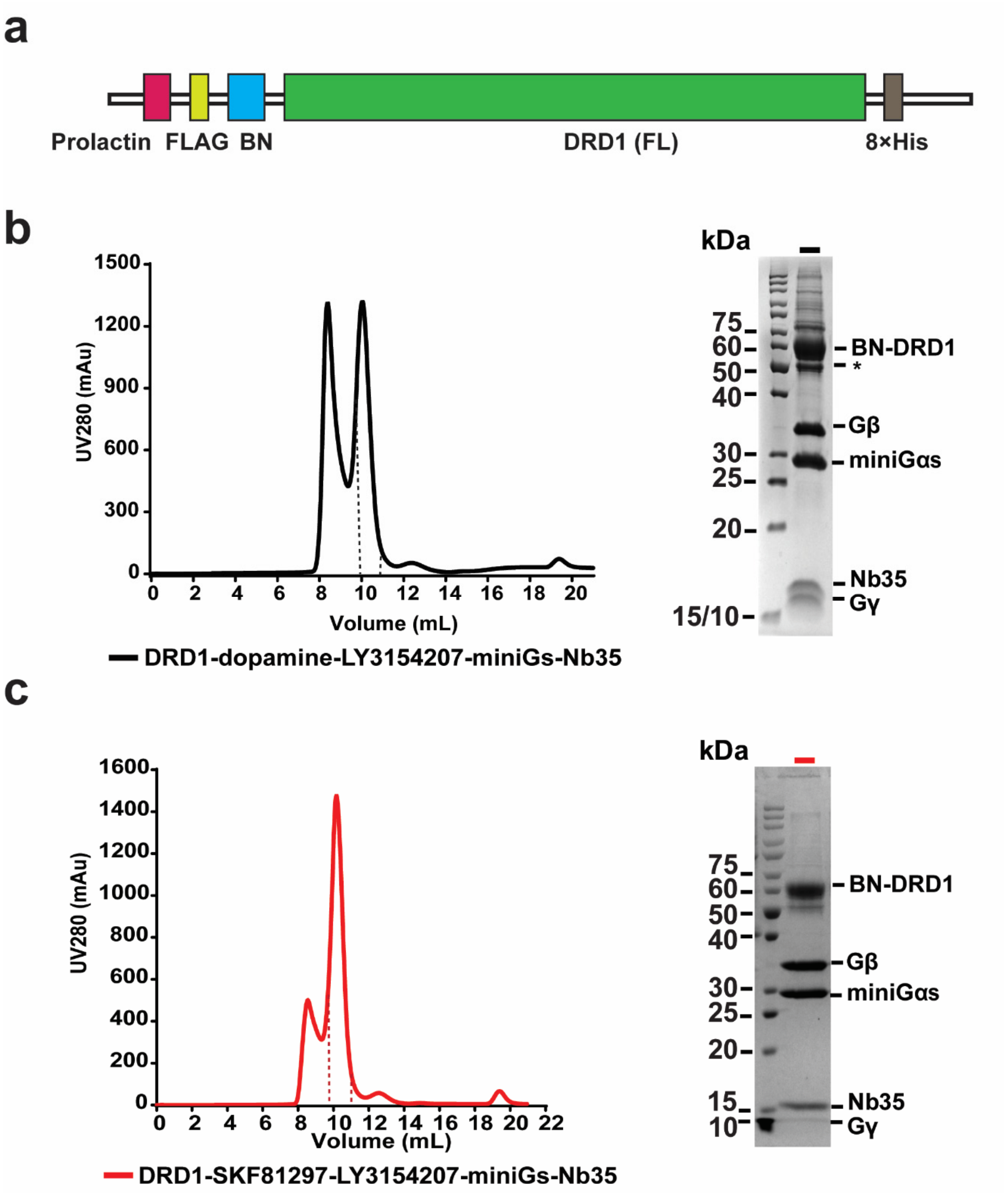

Fig. S2 Construct and preparation of DRD1-miniG$_s$ cryo-EM samples. a A cartoon model of the DRD1 construct used for cryo-EM studies. BN: β_2AR N-terminal tail region; Prolactin: prolactin signal peptide sequence. **b** Size exclusion chromatography profile and SDS-PAGE analysis of DRD1-dopamine/LY3154207-miniG$_s$ complex. Fractions between

the two dashed line were collected and concentrated for further cryo-EM grid preparation. **c** Size exclusion chromatography separation and SDS-PAGE analysis of the DRD1-SKF81297/LY3154207-miniG$_s$ complex. Protein fractions between the two dashed line were collected and concentrated for subsequent cryo-EM studies.

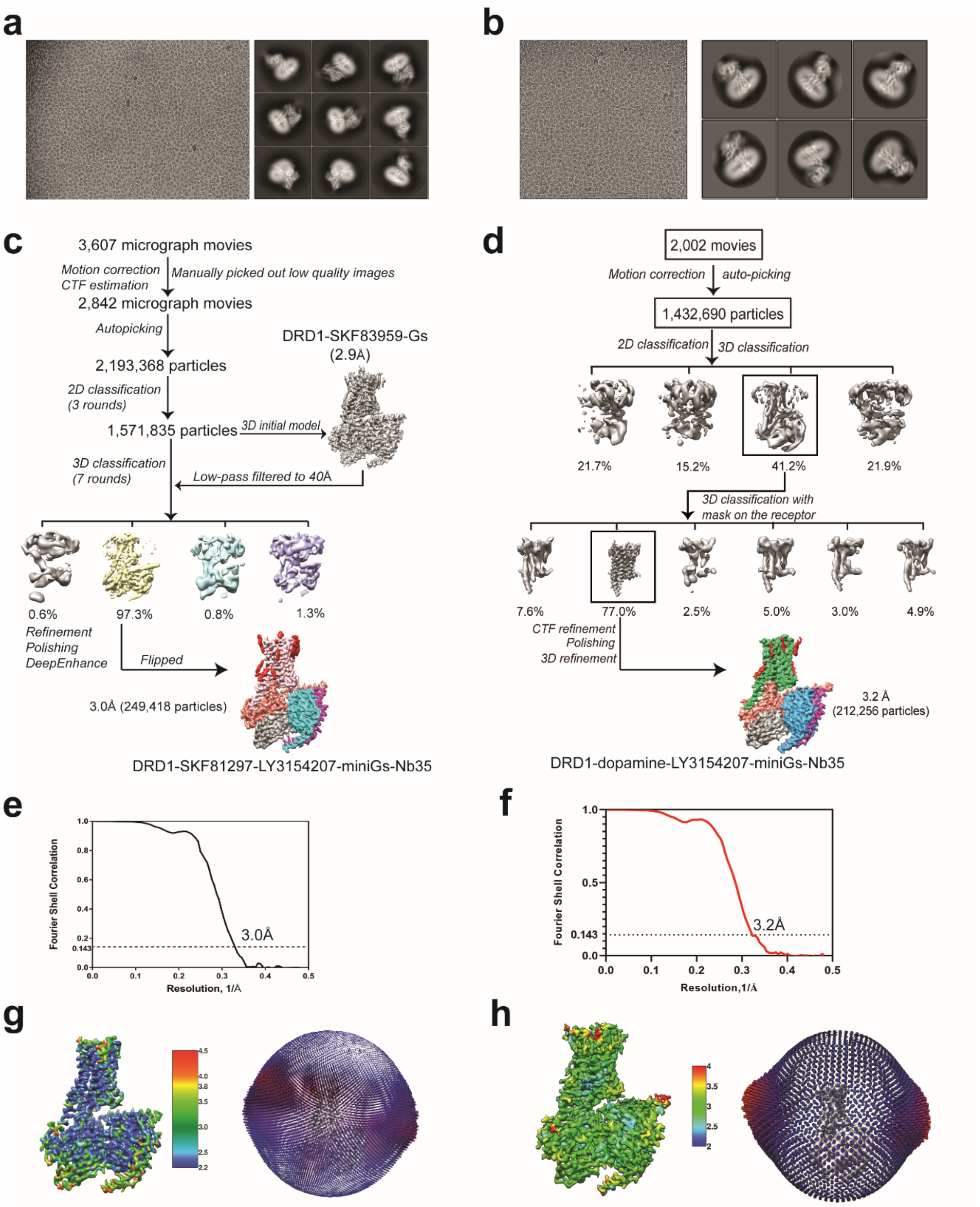

Fig. S3 Single particle cryo-EM workflows for structure determination of DRD1-miniG$_s$ complexes. a, b Representative cryo-EM micrographs and 2D classification averages of DRD1-SKF81297/LY3154207-miniG$_s$ **(a)** and DRD1-dopamine/LY3154207-

miniG$_s$ **(b)**. The 2D averages show divergent secondary structural features from different views. **c-f** Cryo-EM data processing flowcharts of DRD1-SKF81297/LY3154207-miniG$_s$ **(c)** and DRD1-dopamine/LY3154207-miniG$_s$ **(d)** by Relion 3.0, including the final processed global density maps and the 'Gold-standard' Fourier shell correlation (FSC) curves of DRD1-SKF81297/LY3154207-miniG$_s$ **(e)** and DRD1-dopamine/LY3154207-miniG$_s$ **(f)**, respectively. The global resolution defined at the FSC=0.143 is 3.0 Å for DRD1-SKF81297/LY3154207-miniG$_s$ and 3.2 Å for DRD1-dopamine/LY3154207-miniG$_s$. **g, h** The global density maps of DRD1-SKF81297/LY3154207-miniG$_s$ **(g)** and DRD1-dopamine/LY3154207-miniG$_s$ **(h)** colored by local resolution (Å), along with the angle distribution maps of particle orientations. The density maps are shown at thresholds of 0.083 and 0.045 for SKF81297/LY3154207 and dopamine/LY3154207-bound complexes, respectively.

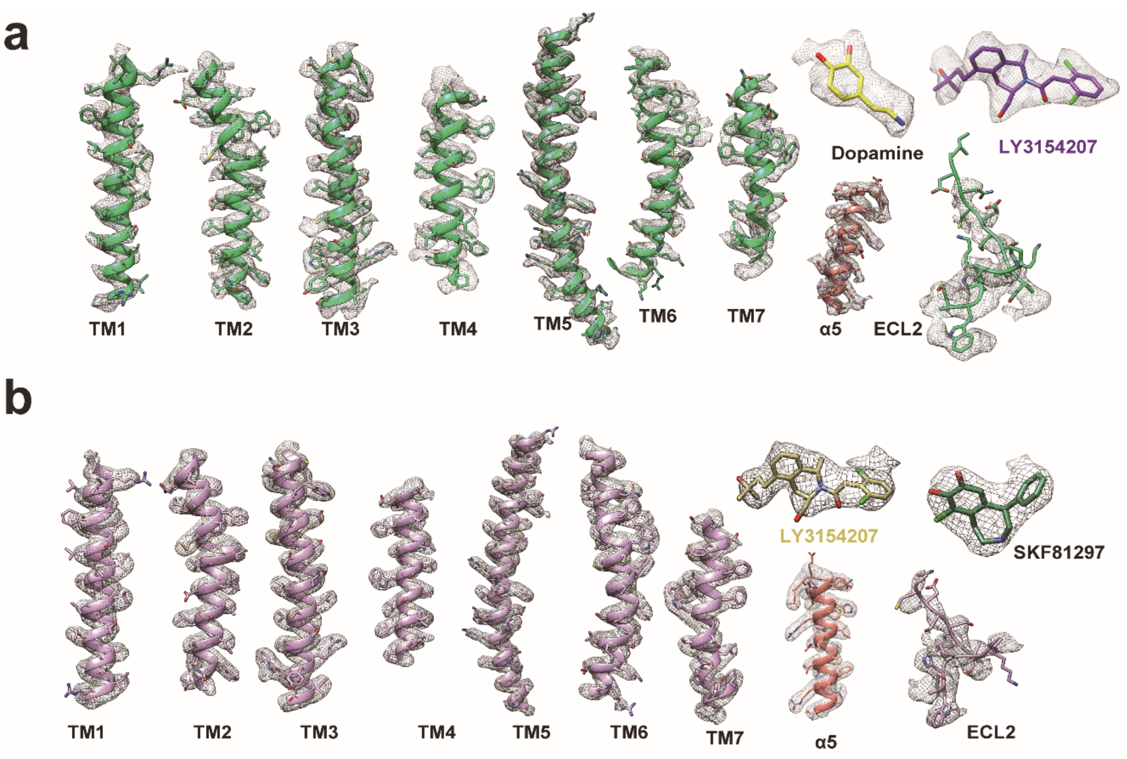

Fig. S4 Local cryo-EM density maps of DRD1-miniG$_s$ complexes. Cryo-EM density maps of TM1–TM7, ligands and ECL2 of DRD1 and α5 helix of miniG$_{\alpha s}$ in the DRD1-dopamine/LY3154207-miniG$_s$ structure **(a)** and the DRD1-SKF81297/LY3154207-miniG$_s$ structure **(b)**.

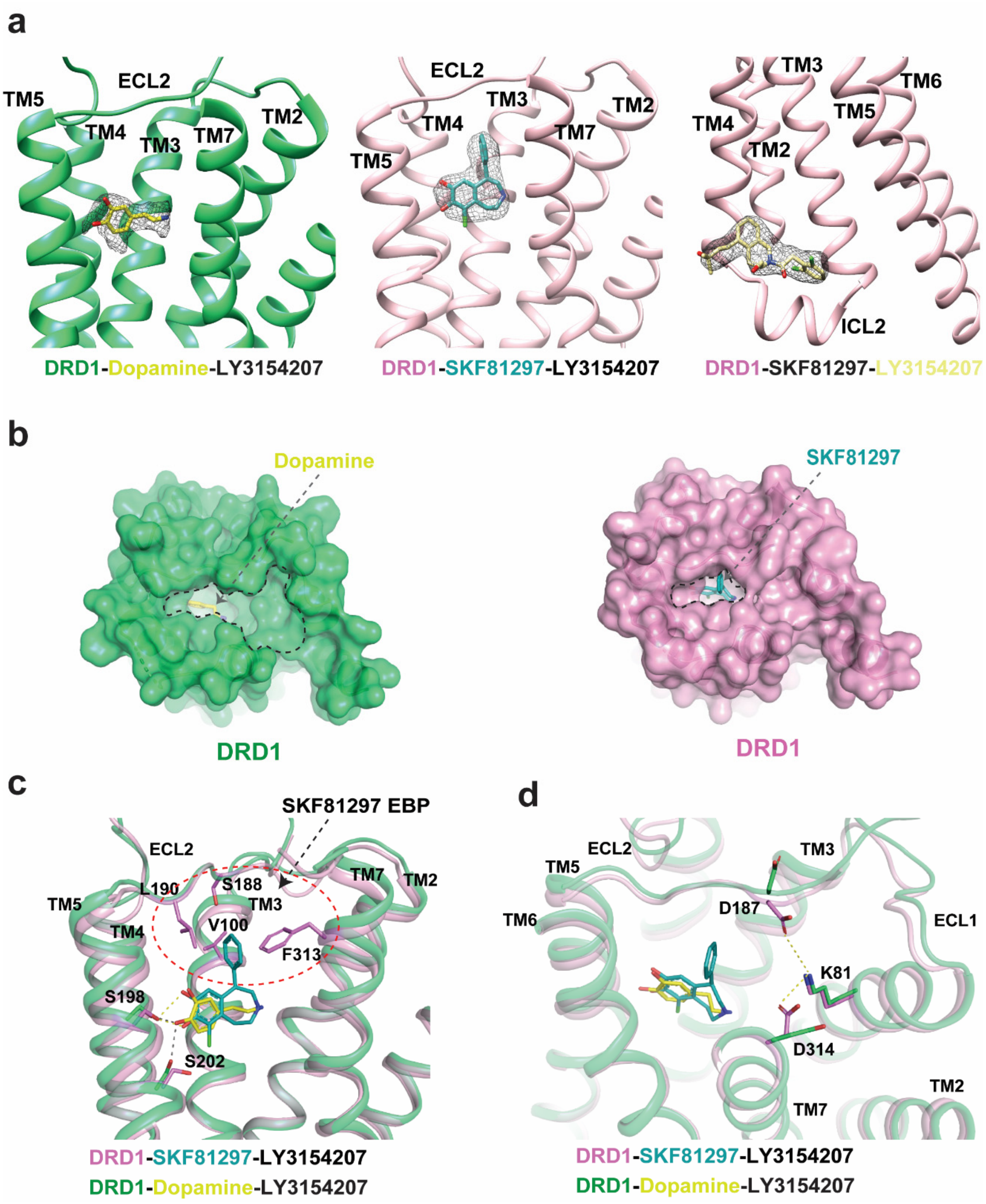

Fig. S5 Topologies of ligand binding pockets of DRD1-miniG$_s$ complexes. a The ligand binding poses of dopamine in the DRD1-dopamine/LY3154207-miniG$_s$ structure and of SKF81297 and LY3154207 in the DRD1-SKF81297/LY3154207-miniG$_s$ structure. The

density of each ligand was clear and shown as mesh. **b** Topologies of extracellular vestibules of DRD1 activated by dopamine and SKF81297, respectively. The dopamine-bound DRD1 adopts a more open extracellular vestibules compared to the SKF81297-bound DRD1. **c** Comparison of interactions and binding poses in the DRD1 ligand binding pockets of dopamine and SKF81297. The SKF81297 compound forms extended interactions with the EBP of DRD1, which is mainly formed by residues from TM3, ECL2 and TM7. The EBP region is circled with a red dashed line. Hydrogen bonds are shown as black dashed lines in the dopamine-bound DRD1 structure; in SKF81297-bound DRD1, the hydrogen bond interactions are shown as yellow dashed line. **d** Comparison of conformational arrangements between residues K81, D187 and D314 in the DRD1-dopamine structure and the DRD1-SKF81297/LY3154207 structure. D187 points toward K81 and forms a polar interaction network with K81 and D314 in the DRD1-SKF81297/LY3154207 structure, but not in the DRD1-dopamine/LY3154207 structure. The polar interactions are shown as yellow dashed lines.

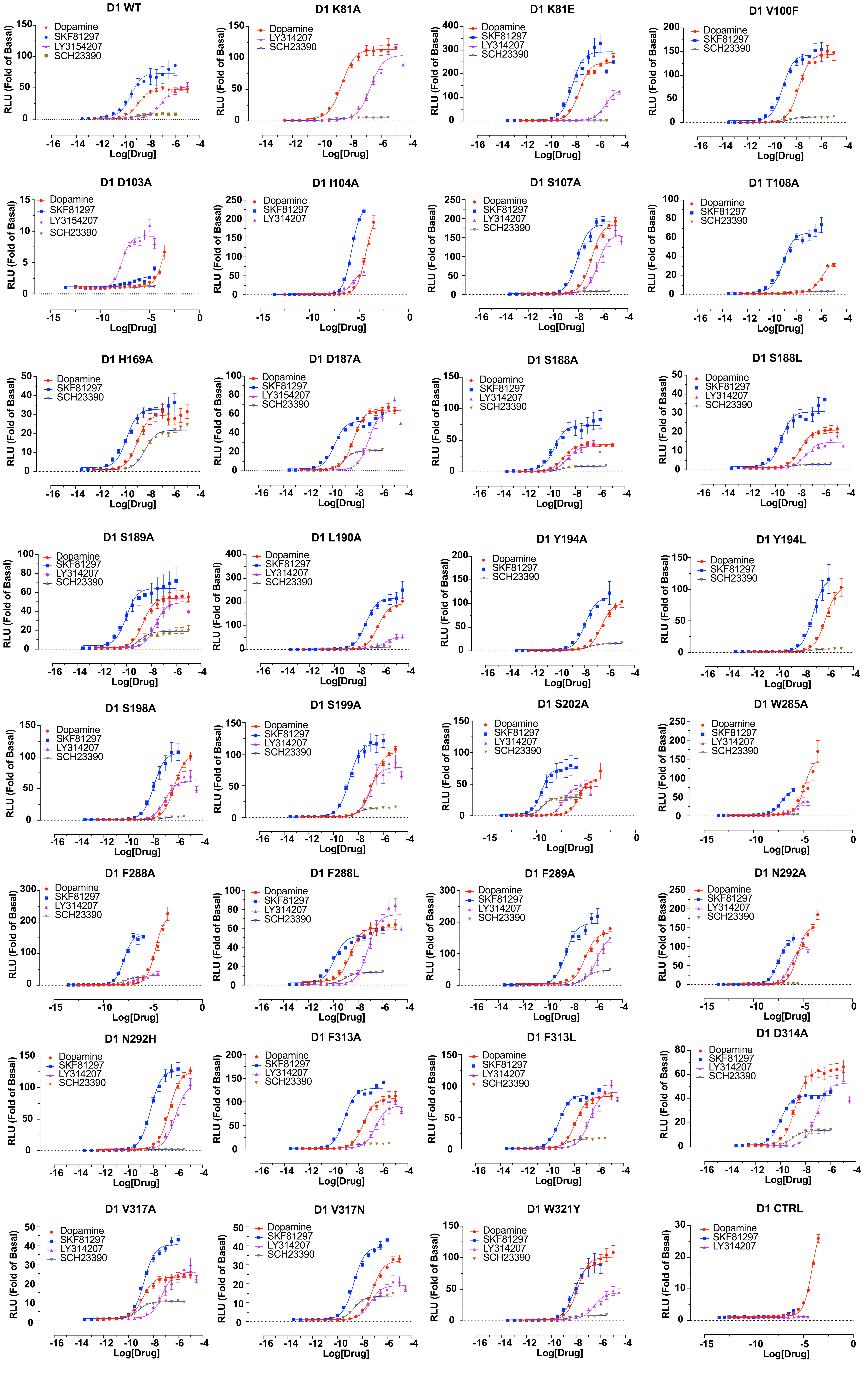
D1 WT
Dopamine
SKF81297
LY3154207
SCH23390
RLU (Fold of Basal)
Log[Drug]
D1 K81A
Dopamine
LY314207
SCH23390
D1 K81E
Dopamine
SKF81297
LY314207
SCH23390
D1 V100F
Dopamine
SKF81297
SCH23390
D1 D103A
Dopamine
SKF81297
LY3154207
SCH23390
D1 I104A
Dopamine
SKF81297
LY314207
D1 S107A
Dopamine
SKF81297
LY314207
SCH23390
D1 T108A
Dopamine
SKF81297
SCH23390
D1 H169A
Dopamine
SKF81297
SCH23390
D1 D187A
Dopamine
SKF81297
LY3154207
SCH23390
D1 S188A
Dopamine
SKF81297
LY314207
SCH23390
D1 S188L
Dopamine
SKF81297
LY314207
SCH23390
D1 S189A
Dopamine
SKF81297
LY314207
SCH23390
D1 L190A
Dopamine
SKF81297
LY314207
SCH23390
D1 Y194A
Dopamine
SKF81297
SCH23390
D1 Y194L
Dopamine
SKF81297
SCH23390
D1 S198A
Dopamine
SKF81297
LY314207
SCH23390
D1 S199A
Dopamine
SKF81297
LY314207
SCH23390
D1 S202A
Dopamine
SKF81297
LY314207
SCH23390
D1 W285A
Dopamine
SKF81297
LY314207
SCH23390
D1 F288A
Dopamine
SKF81297
LY314207
SCH23390
D1 F288L
Dopamine
SKF81297
LY314207
SCH23390
D1 F289A
Dopamine
SKF81297
LY314207
SCH23390
D1 N292A
Dopamine
SKF81297
LY314207
SCH23390
D1 N292H
Dopamine
SKF81297
LY314207
SCH23390
D1 F313A
Dopamine
SKF81297
LY314207
SCH23390
D1 F313L
Dopamine
SKF81297
LY314207
SCH23390
D1 D314A
Dopamine
SKF81297
LY314207
SCH23390
D1 V317A
Dopamine
SKF81297
LY314207
SCH23390
D1 V317N
Dopamine
SKF81297
LY314207
SCH23390
D1 W321Y
Dopamine
SKF81297
LY314207
SCH23390
D1 CTRL
Dopamine
SKF81297
LY314207

Fig. S6 Comparison of dopamine, SKF81297, and LY3154207 in cAMP accumulation assays with WT DRD1 and DRD1 mutants. Normalized dose response curves of cAMP accumulation assay (Glosensor) data of WT DRD1 and DRD1 mutants activated by dopamine, SKF81297 and LY3154207, respectively. Data are presented as means ± SEM with a minimum of two technical replicates and n = 3 biological replicates.

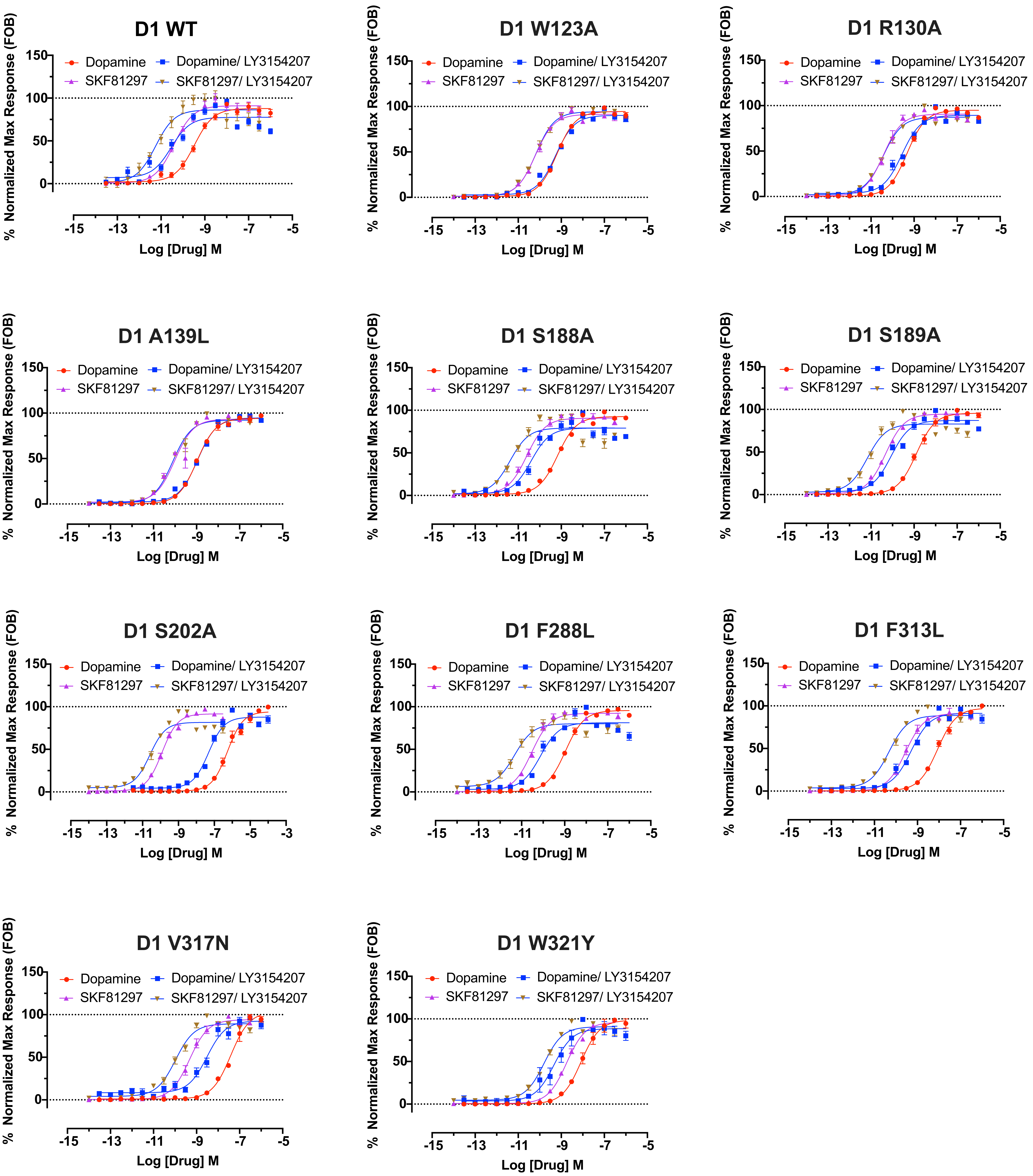
D1 WT
D1 W123A
D1 R130A
D1 A139L
D1 S188A
D1 S189A
D1 S202A
D1 F288L
D1 F313L
D1 V317N
D1 W321Y
Dopamine
Dopamine/ LY3154207
SKF81297
SKF81297/ LY3154207
% Normalized Max Response (FOB)
Log [Drug] M
150
100
50
0
-15
-13
-11
-9
-7
-5
-3

Fig. S7 Comparison of dopamine and SKF81297 cAMP accumulation assays to WT DRD1 and DRD1 mutants in the presence or absence of 30 nM LY3154207. Data are presented as means ± SEM with a minimum of two technical replicates and *n* = 3 biological replicates. F-test analysis of pEC50 values of the following mutants were not statistically different when comparing dose response curves in the presence and absence of 30 nM LY3154207 indicative of diminished allosteric effect at these mutants. F-test analysis ($P < 0.05$), $F_{(1,258)}W123A^{Dopamine} = 1.263$, $P = 0.2622$; $F_{(1,168)}W123A^{SKF81297} = 0.1890$, $P = 0.6643$; $F_{(1,279)}R130A^{SKF81297} = 0.02710$, $P = 0.8694$; $F_{(1,258)}A139L^{Dopamine} = 0.5500$, $P = 0.4591$; $F_{(1,279)}A139L^{SKF81297} = 1.525$, $P = 0.2180$. WT F-test analysis for comparison which was statistically different, $F_{(1,362)}WT^{Dopamine} = 113.5$, $P < 0.0001$ and $F_{(1,354)}WT^{SKF81297} = 36.11$, $P < 0.0001$. See Supplementary information, Table S3 for additional statistical information. Data related to Fig. 1g and 1h.

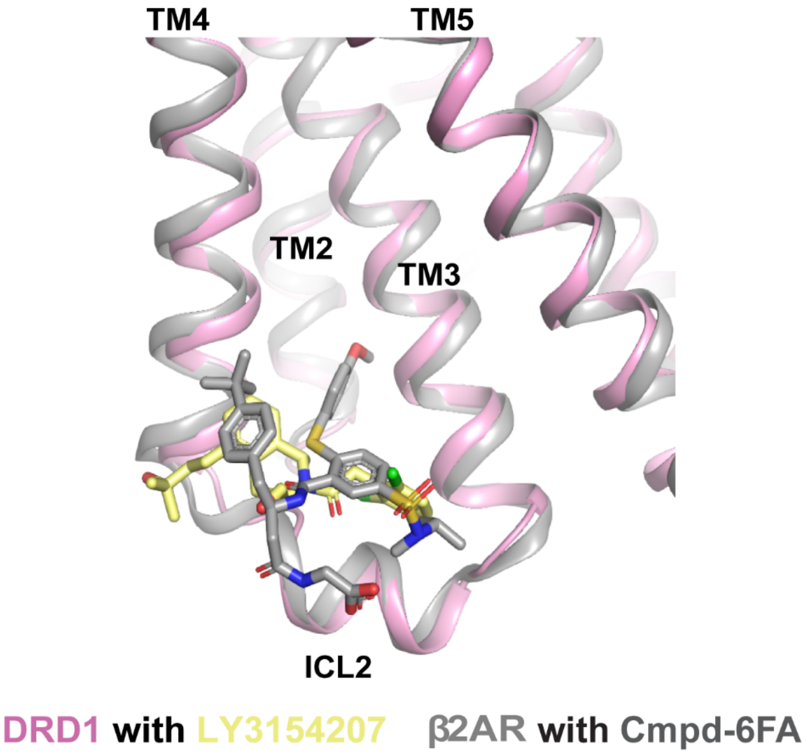

Fig. S8 Structural alignment of the PAM-binding sites in DRD1 and β$_2$AR. The structure of β$_2$AR bound with Cmpd-6FA (PDB code: 6N48) is colored gray, DRD1 is colored pink and the DRD1 PAM compound LY3154207 is colored light yellow.

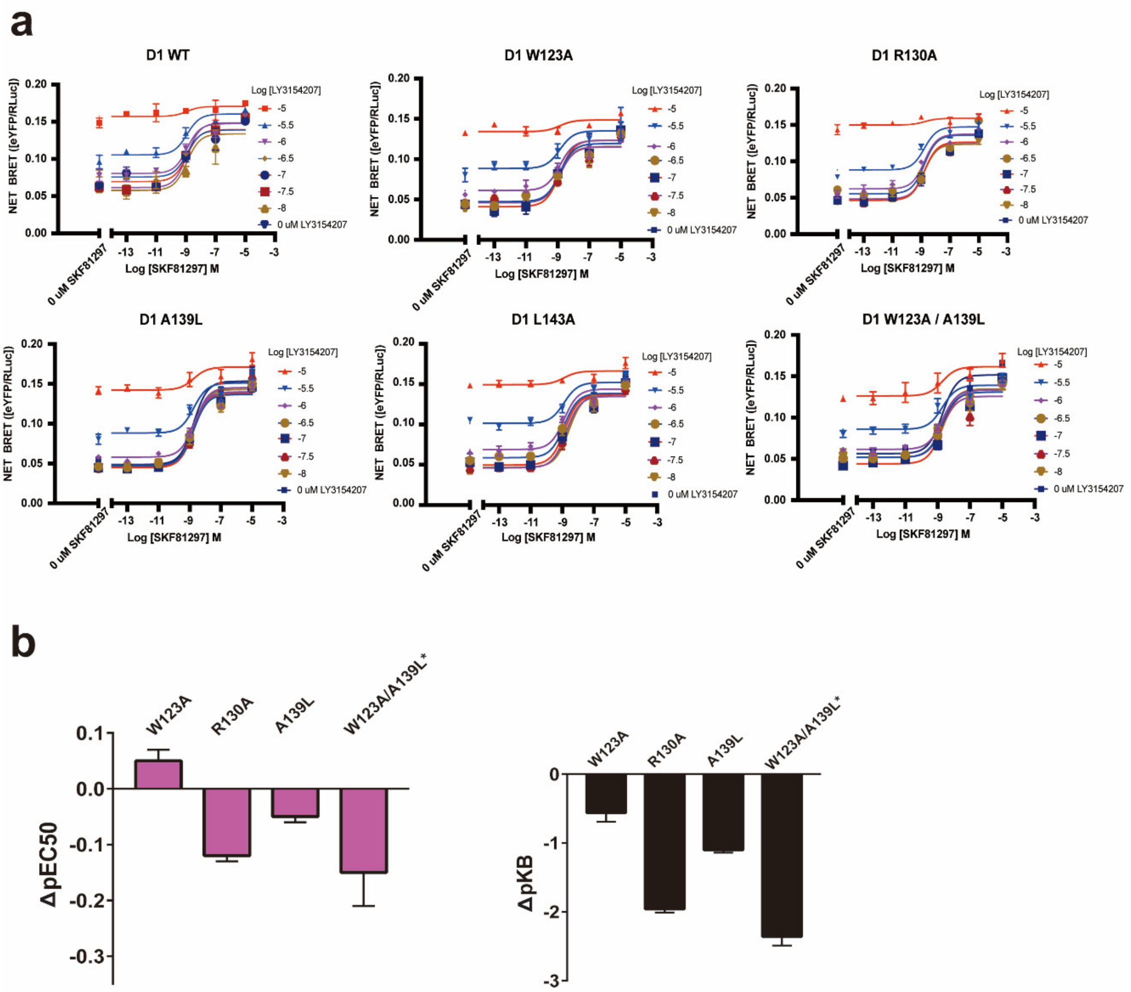

Fig. S9 Mutations of D1R allosteric site residues affect LY3154207 allosteric properties. BRET Venus-MiniG_s recruitment SKF81297 and LY3154207 dose response curves **(a)** and bar charts **(b)** for identified D1R WT and mutations. F-test analysis ($P < 0.05$) comparing 0 µM and 30 nM ($\log_{10} = -7.5$) LY3154207 show only WT is statistically different indicating an allosteric effect for WT and diminished allosteric effect for identified allosteric site mutants. F-test results - $F_{(3,63)}$WT = 5.798, P = 0.0014; $F_{(3,63)}$W123A = 1.380, P = 0.2572; $F_{(3,63)}$R130A = 0.4484, P = 0.7193; $F_{(3,63)}$A139L = 0.8458, P = 0.4740; $F_{(3,63)}$L143A = 1.814, P = 0.1536; $F_{(3,38)}$W123A/A139L = 1.873, P = 0.1507.

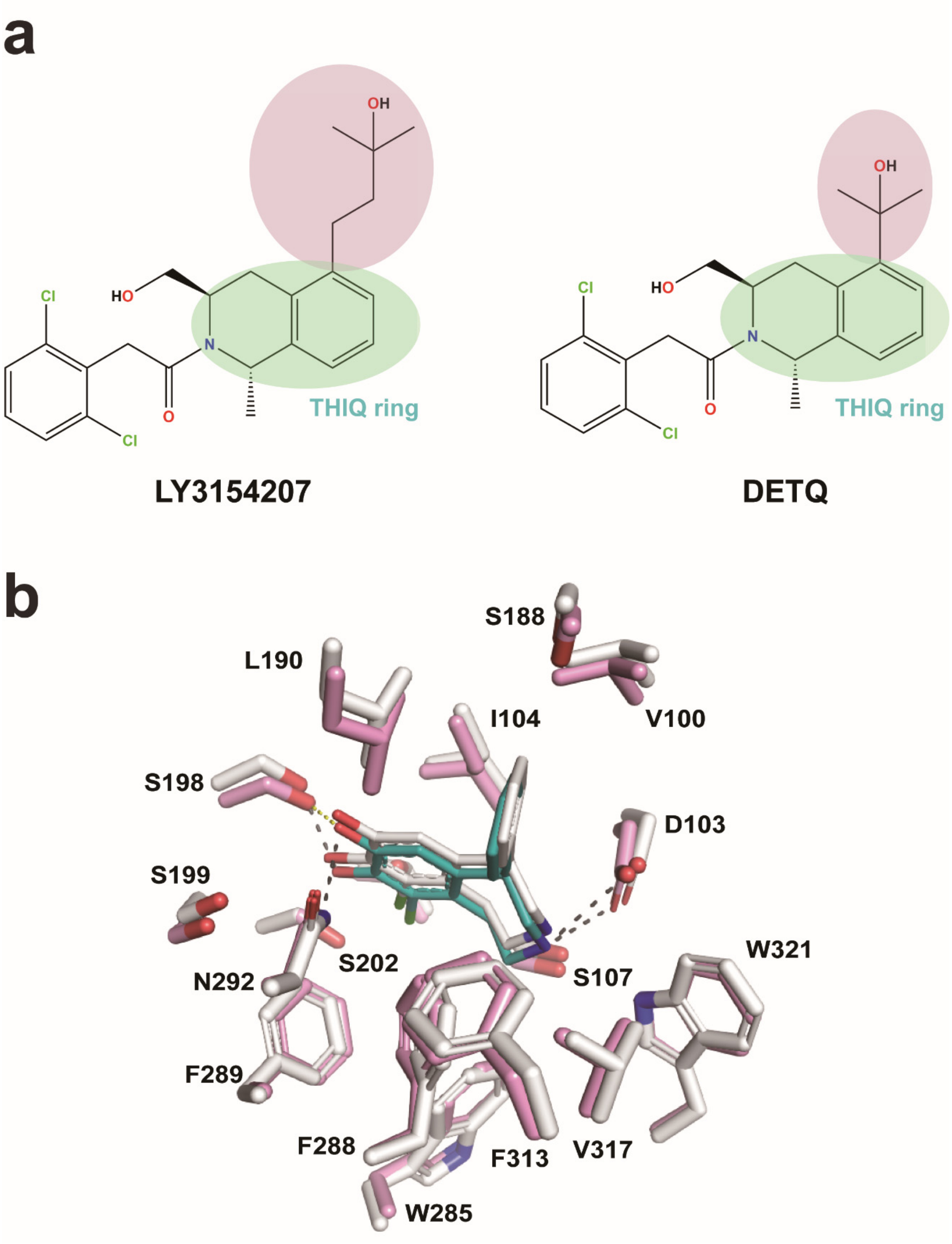

Fig. S10 Superposition of the orthosteric pocket in active DRD1 in the presence or absence of LY3154207. a Chemical structures of DRD1 PAM compounds DETQ and

LY3154207. The THIQ ring is marked with a light green circle while the alkyl linker between the C5 tertiary alcohol and THIQ ring and C5 tertiary alcohol is marked with a light pink circle in both structures. The alkyl linker in LY3154207 is longer than that of DETQ, which is the only difference between these two DRD1 PAMs. **b** Preserved SKF81297 orthosteric binding pocket in active DRD1 with or without LY3154207. The residues participating in interaction with agonist are well overlapped while the binding pose of SKF81297 in LY3154207-bound active DRD1, which is deeper than that of active DRD1 alone, leading to a closer polar interaction with S202. The DRD1-SKF81297 structure without PAM is colored white, the DRD1 and SKF81297 in the DRD1-SKF81297/LY3154207 structure ware colored pink and steal, respectively. The shared hydrogen bond interactions are shown as black dashed line while the additional hydrogen interaction between $S198^{5.42}$ and SKF81297 in the DRD1-SKF81297/LY3154207 structure is shown as yellow dashed line.

Table S1 Cryo-EM data collection, refinement and validation statistics.

	DRD1-miniG$_s$	
	**SKF81297+LY3154207**	**Dopamine+LY3154207**
**Data collection and processing**		
**Magnification**	81,000	49,310
**Voltage (kV)**	300	300
**Electron exposure (e⁻/Å²)**	80	64
**Defocus range (μm)**	-0.5 ~ -3.0	-0.5 ~ -3.0
**Pixel size (Å)**	1.045	1.014
**Symmetry imposed**	C1	C1
**Initial particle projections (no.)**	2,193,368	1,432,690
**Final particle projections (no.)**	249,418	212,256
**Map resolution (Å)**	3.0	3.2
**FSC threshold**	0.143	0.143
**Map resolution range (Å)**	2.3-4.3	2.5-5.0
**Refinement**		
**Initial model used (PDB code)**	7JV5	7JV5
**Model resolution (Å)**	3.0	3.2
**FSC threshold**	0.143	0.143
**Model resolution range (Å)**	50-3.0	50-3.2
**Map sharpening *B* factor (Å²)**	-123.43	-114.48
**Model composition**		
**Non-hydrogen atoms**	8544	8471
**Protein residues**	1040	1044
**Ligand**	2	2
**Lipids**	12	9
**Water**	0	0
***B* factors (Å²)**		
**Protein**	65.1	71.2
**Ligand**	57.0	49.2
**Lipids**	138.5	80.8
**Water**	n/a	n/a
**R.m.s. deviations**		
**Bond lengths (Å)**	0.009	0.004
**Bond angles (°)**	1.362	0.85
**Validation**		
**MolProbity score**	1.39	1.17
**Clashscore**	4.61	3.77
**Rotamer outliers (%)**	0.22	0.0
**Ramachandran plot**		
**Favored (%)**	97.17	98.04
**Allowed (%)**	2.83	1.96
**Disallowed (%)**	0	0

Table S2 G_S-mediated cAMP accumulation results of WT DRD1 and DRD1 mutants in orthosteric site.

D1R	Dopamine				SKF81297				LY3154207				SCH23390			
	Fold of Basal (FOB)	ΔFOB	pEC50	ΔpEC50	Fold of Basal (FOB)	ΔFOB	pEC50	ΔpEC50	Fold of Basal (FOB)	ΔFOB	pEC50	ΔpEC50	Fold of Basal (FOB)	ΔFOB	pEC50	ΔpEC50
WT	51.93 ± 7.01	0.00	9.12 ± 0.16	0.00	74.57 ± 30.15	0.00	9.85 ± 0.28	0.00	53.34 ± 13.1	0.00	7.02 ± 0.28	0.00	9.47 ± 1.24	0.00	9.04 ± 0.13	0.00
K81A	118.73 ± 19.08	66.80	8.72 ± 0.23	-0.40	ND	ND	ND	ND	107.97 ± 10.05	54.63	6.7 ± 0.4	-0.32	5.45 ± 0.24	-4.01	8.85 ± 0.08	-0.20
K81E	262.73 ± 28.82	210.80	7.69 ± 0.18	-1.43	286.33 ± 97.96	211.76	8.35 ± 0.28	-1.50	192.81 ± 41.47	139.47	5.09 ± 0.59	-1.94	2.7 ± 0.54	-6.77	8.17 ± 0.03	-0.87
V100F	192.7 ± 69.49	140.77	8.09 ± 0.21	-1.03	137.85 ± 39.77	63.28	9.07 ± 0.22	-0.78	ND	ND	ND	ND	9.98 ± 1.02	0.51	8.61 ± 0.14	-0.43
D103A	15.82 ± 5.73	-36.11	3.32 ± 0.04	-5.81	7.05 ± 3.4	-67.52	4.77 ± 1.13	-5.08	9.54 ± 1.83	-43.80	7.73 ± 0.21	0.71	ND	ND	ND	ND
I104A	317.7 ± 53.92	265.77	4.13 ± 0.19	-4.99	297.19 ± 57.92	222.62	5.86 ± 0.14	-3.99	83.09 ± 60.02	29.75	6.23 ± 0.92	-0.79	ND	ND	ND	ND
S107A	200.52 ± 24.88	148.59	6.89 ± 0.16	-2.23	188.29 ± 44.68	113.73	8.27 ± 0.25	-1.58	167.08 ± 26.73	113.74	6.23 ± 0.28	-0.79	7.64 ± 0.62	-1.82	8.61 ± 0.06	-0.44
T108A	29.7 ± 9.4	-22.23	5.85 ± 0.19	-3.27	68.57 ± 17.33	-6.00	8.76 ± 0.19	-1.09	ND	ND	ND	ND	3.43 ± 0.52	-6.03	8.91 ± 0.12	-0.13
H169A	30.03 ± 7.91	-21.90	9.11 ± 0.24	-0.01	34.17 ± 9.77	-40.40	10.05 ± 0.32	0.20	ND	ND	ND	ND	21.33 ± 2.3	11.86	8.3 ± 0.07	-0.74
D187A	65.11 ± 6.61	13.18	8.35 ± 0.13	-0.77	ND	ND	ND	ND	66.84 ± 3.56	13.50	6.94 ± 0.32	-0.08	21.93 ± 2.11	12.46	8.96 ± 0.12	-0.09
S188A	43.46 ± 4.6	-8.47	9.05 ± 0.16	-0.07	54.34 ± 21.6	-20.22	10.17 ± 0.26	0.32	39.81 ± 5.12	-13.53	7.03 ± 0.22	0.01	9.18 ± 1.22	-0.29	8.92 ± 0.16	-0.12
S188L	21.95 ± 3.81	-29.98	7.98 ± 0.2	-1.14	25.63 ± 5.63	-48.94	9.76 ± 0.4	-0.09	15.69 ± 3.29	-37.65	7.44 ± 0.22	0.42	3.17 ± 0.18	-6.30	8.32 ± 0.37	-0.73
S189A	59.72 ± 13.41	7.79	8.66 ± 0.11	-0.46	70.18 ± 29.28	-4.39	10.14 ± 0.32	0.29	51.75 ± 2.51	-1.59	7.05 ± 0.47	0.03	19.86 ± 6.77	10.40	8.92 ± 0.14	-0.12
L190A	258.06 ± 31.62	206.13	6.39 ± 0.49	-2.73	220.89 ± 40.72	146.32	7.47 ± 0.08	-2.38	89.88 ± 34.83	36.54	5.5 ± 1.08	-1.52	10.43 ± 0.88	0.96	7.34 ± 0.14	-1.70
Y194A	113.57 ± 18.62	61.64	6.6 ± 0.15	-2.53	117.01 ± 43.76	42.44	8.11 ± 0.33	-1.74	ND	ND	ND	ND	12.72 ± 0.96	3.25	7.56 ± 0.05	-1.48
Y194L	124.91 ± 45.48	72.98	6.32 ± 0.33	-2.80	133.99 ± 70.23	59.42	7.38 ± 0.28	-2.47	ND	ND	ND	ND	4.91 ± 0.67	-4.56	7.41 ± 0.23	-1.63
S198A	94.25 ± 23.63	42.32	6.09 ± 0.31	-3.03	98.06 ± 40.31	23.49	8.11 ± 0.19	-1.74	63.98 ± 16.46	10.64	6.99 ± 0.25	-0.03	5.97 ± 0.79	-3.50	7.07 ± 0.1	-1.97
S199A	107.63 ± 6.91	55.69	6.86 ± 0.07	-2.26	116.04 ± 28.56	41.47	8.76 ± 0.15	-1.09	84.11 ± 23.54	30.77	6.78 ± 0.54	-0.25	16.06 ± 2.47	6.59	8.01 ± 0.17	-1.04
S202A	67.82 ± 15.78	15.89	5.46 ± 0.32	-3.66	73.94 ± 32.29	-0.63	9.59 ± 0.18	-0.26	48.37 ± 7.65	-4.97	7.09 ± 0.23	0.07	29.25 ± 7.07	19.78	9.39 ± 0.12	0.34
W285A	368.54 ± 143.29	316.61	4.43 ± 0.33	-4.69	ND	ND	ND	ND	72.48 ± 59.8	19.14	6.25 ± 0.88	-0.77	3.6 ± 0.19	-5.87	8.8 ± 0.1	-0.24
F288A	199.38 ± 47.89	147.45	4.56 ± 0.31	-4.56	101.59 ± 33.5	27.03	8.09 ± 0.15	-1.76	67.38 ± 46.64	14.04	6.19 ± 1	-0.83	27.75 ± 6.87	18.28	7.81 ± 0.12	-1.24
F288L	66.52 ± 11.33	14.59	8.9 ± 0.43	-0.22	63.1 ± 22.14	-11.47	9.58 ± 0.9	-0.27	86.61 ± 15.05	33.27	6.92 ± 0.43	-0.10	13.52 ± 4.38	4.05	8.88 ± 0.04	-0.16
F289A	174.41 ± 32.53	122.48	7.25 ± 0.4	-1.87	ND	ND	ND	ND	161.09 ± 19.76	107.74	5.86 ± 0.44	-1.16	51.13 ± 11.74	41.67	7.43 ± 0.19	-1.62
N292A	222.7 ± 39.57	170.77	4.73 ± 0.27	-4.39	118.3 ± 29.95	43.74	7.57 ± 0.19	-2.28	112.15 ± 10.71	58.81	6.01 ± 0.45	-1.01	2.45 ± 0.14	-7.01	8.28 ± 0.32	-0.76
N292H	120.63 ± 18.62	68.70	6.78 ± 0.14	-2.34	108.49 ± 46.96	33.92	8.35 ± 0.12	-1.50	110.31 ± 0.61	56.97	6 ± 0.5	-1.02	4.6 ± 1.87	-4.86	6.64 ± 1.52	-2.40
F313A	111.41 ± 21.82	59.48	7.62 ± 0.13	-1.50	ND	ND	ND	ND	104.85 ± 15.84	51.51	6.24 ± 0.44	-0.78	11.04 ± 1.64	1.57	8.83 ± 0.05	-0.22
F313L	85.37 ± 13.06	33.44	7.89 ± 0.1	-1.23	79.86 ± 13.51	5.30	9.29 ± 0.12	-0.56	102.7 ± 3.54	49.36	6.44 ± 0.39	-0.58	16.76 ± 2.93	7.29	8.72 ± 0.05	-0.32
D314A	56.68 ± 5.97	4.75	7.38 ± 0.09	-1.75	ND	ND	ND	ND	61.34 ± 19.33	8.00	7 ± 0.29	-0.02	7.4 ± 1.24	-2.07	9.14 ± 0.09	0.10
V317A	23.8 ± 4.06	-28.13	8.89 ± 0.12	-0.24	41.76 ± 4.14	-32.80	8.7 ± 0.03	-1.16	27.76 ± 7.1	-25.58	7.08 ± 0.28	0.06	10.39 ± 0.49	0.93	9.17 ± 0.06	0.13
V317N	34.13 ± 4.23	-17.80	7.11 ± 0.15	-2.01	35.39 ± 4.01	-39.18	8.72 ± 0.1	-1.13	19.77 ± 6.35	-33.57	7.37 ± 0.19	0.34	14.68 ± 0.85	5.21	8.47 ± 0.06	-0.57
W321Y	105.53 ± 22.86	53.60	7.86 ± 0.2	-1.26	118.44 ± 32.63	43.87	8.48 ± 0.18	-1.37	51.3 ± 15.73	-2.04	6.37 ± 0.04	-0.66	7.54 ± 1.57	-1.93	8.14 ± 0.15	-0.90

Data are presented as means ± SEM with a minimum of two technical replicates and *n* = 3 biological replicates. Greek letter delta (Δ) for the difference (ΔpEC50) and Fold of Basal (ΔFOB) when compared with the wild-type receptor values. ND, Not Determined. Data related to Supplementary information, Fig. S6.

Table S3 G_S-mediated cAMP accumulation analysis of WT DRD1 and DRD1 mutants with or without PAM LY3154207.

DRD1	Dopamine		Dopamine/LY3154207			SKF81297		SKF81297/LY3154207		
	pEC50	ΔpEC50	pEC50	ΔpEC50	pK_B	pEC50	ΔpEC50	pEC50	ΔpEC50	pK_B
**WT**	9.49 ± 0.06	0	10.53 ± 0.11	1.04	8.11 ± 0.16	10.41 ± 0.06	0	11.24 ± 0.12	0.83	8.51 ± 0.33
**W123A**	9.20 ± 0.06	-0.29	9.24 ± 0.07	-0.25	6.03 ± 0.41	10.22 ± 0.08	-0.19	10.19 ± 0.09	-0.22	6.18 ± 0.34
**R130A**	9.33 ± 0.06	-0.16	9.60 ± 0.09	0.11	7.72 ± 0.46	10.44 ± 0.07	0.03	10.46 ± 0.09	0.05	6.11 ± 0.24
**A139L**	9.07 ± 0.05	-0.42	8.97 ± 0.06	-0.52	6.57 ± 0.50	9.97 ± 0.09	-0.44	10.11 ± 0.10	-0.31	6.03 ± 0.25
**S188A**	9.17 ± 0.10	-0.32	10.48 ± 0.24	0.99	8.24 ± 0.42	10.72 ± 0.05	0.29	11.45 ± 0.16	1.04	8.71 ± 0.53
**S189A**	9.01 ± 0.09	-0.48	10.22 ± 0.16	0.73	7.93 ± 0.30	10.38 ± 0.07	-0.03	11.21 ± 0.16	0.80	8.63 ± 0.44
**S202A**	5.92 ± 0.04	-3.57	7.03 ± 0.10	-2.46	8.45 ± 0.30	9.85 ± 0.06	-0.56	10.61 ± 0.13	0.20	8.16 ± 0.25
**F288L**	8.88 ± 0.06	-0.61	10.25 ± 0.10	0.76	7.90 ± 0.16	10.53 ± 0.06	0.12	11.27 ± 0.15	0.86	8.58 ± 0.42
**F313L**	8.11 ± 0.04	-1.38	9.24 ± 0.08	0.25	8.14 ± 0.12	9.50 ± 0.06	-0.91	10.29 ± 0.10	-0.12	8.49 ± 0.27
**V317N**	7.40 ± 0.10	-2.09	8.48 ± 0.22	-1.01	8.11 ± 0.32	9.40 ± 0.13	-1.01	10.06 ± 0.20	-0.35	8.51 ± 0.48
**W321Y**	8.10 ± 0.09	-1.39	9.51 ± 0.17	0.02	7.95 ± 0.21	8.76 ± 0.04	-1.65	9.80 ± 0.12	-0.61	8.38 ± 0.19

Data are presented as means ± SEM with a minimum of two technical replicates and $n = 3$ biological replicates. Greek letter delta (Δ) for the difference (ΔpEC50) when compared with the wild-type receptor values in the absence of 30 nM LY3154207. Data related to Fig. 1g and 1h.

Table S4 Allosteric effect on WT and mutant DRD1 using G protein recruitment assay.

	SKF81297		SKF81297/LY3154207			
	pEC_{50}	ΔpEC_{50}	pEC_{50}	ΔpEC_{50}	pK_B	ΔpK_B
**WT**	8.79 ± 0.12	0.00	8.94 ± 0.06	0.00	8.04 ± 0.23	0.00
**W123A**	8.84 ± 0.10	0.05	8.95 ± 0.06	0.01	7.48 ± 0.10	-0.56
**R130A**	8.67 ± 0.13	-0.12	8.86 ± 0.05	-0.08	6.08 ± 0.28	-1.96
**A139L**	8.74 ± 0.13	-0.05	8.75 ± 0.06	-0.19	6.94 ± 0.27	-1.10
**L143A**	8.67 ± 0.13	-0.12	8.77 ± 0.06	-0.17	7.90 ± 0.08	-0.14
**W123A/A139L***	8.64 ± 0.18	-0.15	8.71 ± 0.07	-0.23	5.68 ± 0.36	-2.36

MiniGs recruitment using BRET assay and transfected HEKT cells in the presence of increasing concentrations of SKF81297 and LY3154207. Allosteric parameter (pK_B) obtained by fitting dose response curves to "Allosteric EC50 shift" function of Graphpad Prism 8.4. Data are presented as means ± SEM with a minimum of two technical replicates and n = 3 biological replicates. * denoted n = 2 biological replicates. Greek letter delta (Δ) for the difference (ΔpEC50) or affinity (ΔpK_B) when compared with the wild-type receptor values. Data related to Supplementary information, Fig. S9.
